# Mechanosensitive Ion Channel TMEM63A Gangs Up with Local Macrophages to Modulate Chronic Post-amputation Pain

**DOI:** 10.1007/s12264-022-00910-0

**Published:** 2022-07-12

**Authors:** Shaofeng Pu, Yiyang Wu, Fang Tong, Wan-Jie Du, Shuai Liu, Huan Yang, Chen Zhang, Bin Zhou, Ziyue Chen, Xiaomeng Zhou, Qingjian Han, Dongping Du

**Affiliations:** 1 Pain Management Center, Shanghai Jiao Tong University Affiliated Sixth People’s Hospital, Shanghai, 200233 China; 2 State Key Laboratory of Medical Neurobiology and MOE Frontier Center for Brain Science, Institutes of Brain Science, Fudan University, Shanghai, 200032 China

**Keywords:** Mechanosensitive ion channel, TMEM63A, Post-amputation pain, Tibial nerve transfer, Macrophage

## Abstract

Post-amputation pain causes great suffering to amputees, but still no effective drugs are available due to its elusive mechanisms. Our previous clinical studies found that surgical removal or radiofrequency treatment of the neuroma at the axotomized nerve stump effectively relieves the phantom pain afflicting patients after amputation. This indicated an essential role of the residual nerve stump in the formation of chronic post-amputation pain (CPAP). However, the molecular mechanism by which the residual nerve stump or neuroma is involved and regulates CPAP is still a mystery. In this study, we found that nociceptors expressed the mechanosensitive ion channel TMEM63A and macrophages infiltrated into the dorsal root ganglion (DRG) neurons worked synergistically to promote CPAP. Histology and qRT-PCR showed that TMEM63A was mainly expressed in mechanical pain-producing non-peptidergic nociceptors in the DRG, and the expression of TMEM63A increased significantly both in the neuroma from amputated patients and the DRG in a mouse model of tibial nerve transfer (TNT). Behavioral tests showed that the mechanical, heat, and cold sensitivity were not affected in the *Tmem63a*^*-/-*^ mice in the naïve state, suggesting the basal pain was not affected. In the inflammatory and post-amputation state, the mechanical allodynia but not the heat hyperalgesia or cold allodynia was significantly decreased in *Tmem63a*^*-/-*^ mice. Further study showed that there was severe neuronal injury and macrophage infiltration in the DRG, tibial nerve, residual stump, and the neuroma-like structure of the TNT mouse model, Consistent with this, expression of the pro-inflammatory cytokines TNF-α, IL-6, and IL-1β all increased dramatically in the DRG. Interestingly, the deletion of *Tmem63a* significantly reduced the macrophage infiltration in the DRG but not in the tibial nerve stump. Furthermore, the ablation of macrophages significantly reduced both the expression of *Tmem63a* and the mechanical allodynia in the TNT mouse model, indicating an interaction between nociceptors and macrophages, and that these two factors gang up together to regulate the formation of CPAP. This provides a new insight into the mechanisms underlying CPAP and potential drug targets its treatment.

**Supplementary Information:**

The online version contains supplementary material available at 10.1007/s12264-022-00910-0.

## Introduction

Amputation *per se* is a severe physical and psychological traumatic event with a high incidence worldwide. In addition, up to 70%–80% of patients with amputation experience severe chronic post-amputation pain (CPAP), which can be devastating [1, 2]. CPAP is a long-lasting and severe pain after amputation surgery including stump pain and phantom pain. Stump pain can be described as the evoked or spontaneous pain arising from the residual part of the amputated limb [3], while phantom pain can be described as continuous pain sensation arising from the denervated part of the limb that the patients can still feel [4, 5]. Like other canonical pain conditions, allodynia and hyperalgesia are essential components of CPAP. Peripheral sensitization, central sensitization, and cerebral cortical reconstruction have been proposed as possible mechanisms underlying CPAP. However, the responsiveness of CPAP to available painkillers such as the anticonvulsant drug gabapentin and the hormone drug salmon calcitonin is very poor, which indicates that complicated mechanisms are involved [6–12]. Clinical studies have shown that treatments targeting the stump of an amputated peripheral limb can effectively relieve CPAP [13, 14]. Our previous studies had also shown that ablation of the neuroma or the stump of the amputated limb with radiofrequency or surgery significantly improves the pain condition of patients [7, 10], suggesting an essential role of peripheral nerves in the development of CPAP, but the exact mechanisms of how the neuromas or residual parts of peripheral nerves regulate CPAP still need further study.

The mechanosensitive ion channel (MSC) is a subtype of cation channel that converts extracellular mechanical force into an intracellular electrical signal, and elicits a variety of biological processes, such as conscious hearing, touch, and proprioception, as well as non-conscious blood pressure and osmotic pressure [15]. To date, a series of prokaryotic and eukaryotic MSCs have been identified, such as TREK/TRAAK K2P, Piezo1/2, OSCA/TMEM63, and TMC1/2 [16–18]. Recently, increasing evidence has shown that MSCs are also involved in the sensation of pain [19]. DRG neurons that can be labeled with isolectin B4 (IB4) are a subset of high-threshold C-fiber sensory neurons in which noxious mechanical force activates the MSC-like ion channel TCAN to elicit mechanical pain [20]. Two typical phenomena under many different pain conditions are allodynia and hyperalgesia, which are characterized as an innocuous stimulus that evokes pain sensation and a mild pain stimulus that evokes strong pain sensation, respectively. The MSC Piezo 2 has been shown to mediate injury-induced tactile pain hypersensitivity in mice and humans; individuals with loss-of-function mutations in Piezo 2 completely fail to develop sensitization and painful reaction to touch after skin inflammation [19]. The TMEM63 family is a newly-identified osmatic pressure sensation-related MSC in mammals, whose functions are similar to the sonosensitizer Ca^2+^-permeable channel (OSCA) in plants. There are three members, TMEM63A, TMEM63B, TMEM63C, in the TMEM63 family [21]. Studies have shown that these proteins can sense changes in membrane surface tension and play an important role in mechanical signal transduction. Heterozygous missense mutations in TMEM63A result in an infantile disorder resembling a hypomyelinating leukodystrophy [22]. TMEM63B is enriched in the inner ear sensory hair cells, and functions as a cation channel mediating osmosensation and hearing [23]. TMEM63C plays an important role in mediating the glomerular filtration barrier in zebrafish, and patients with TMEM63C loss in podocytes exhibit specific focal segmental glomerulosclerosis [24]. In this study, we found that there was abundant and specific expression of TMEM63A in nonpeptidergic nociceptors. However, the role of TMEM63 in pain sensation has not been determined.

The macrophage is one type of white blood cell of the immune system which acts to defend the host against infection and injury *via* phagocytosis. Macrophage infiltration of the peripheral nervous system has been identified under neuropathic pain conditions [25–29]. Bidirectional interactions between DRG neurons and macrophages are critical for the initiation and maintenance of neuropathic pain [30–33]. Following peripheral nerve injury, dysregulated microRNAs in DRG neurons are released *via* exosomes upon neuronal activity; they are phagocytosed by macrophages and promote a pro-inflammatory phenotype. Simultaneously, the DRG resident macrophages markedly expand and proliferate around injured sensory neurons [29]. TNF-α,IL-1β, and IL-6 secreted from these macrophages potentiate pain hypersensitivity that characterizes the neuropathic pain phenotype [34–42]. An intrathecal anti-IL-6 antibody and IgG attenuate peripheral nerve injury-induced mechanical allodynia in the rat: possible immune modulation of neuropathic pain [43, 44].

Here, we found that the mechanosensitive ion channel TMEM63A was specifically expressed in the mechanical pain-producing non-peptidergic DRG neurons, and the expression level of this MSC was increased significantly in the neuromas of CPAP patients and the DRG of TNT mouse model, which mimics amputation in patients. Further study showed that TMEM63A played an essential role in the development of CPAP through the mediation of macrophage infiltration and pro-inflammatory cytokine release. In addition, the macrophages infiltrated into the residual tibial nerve and injured DRG, which, in turn, promoted the expression of TMEM63A in the DRG and led to more macrophage infiltration and more severe pain hypersensitivity.

## Materials and Methods

### Human Sample and Ethics

This study was approved by the Ethics Committee of Shanghai Sixth People’s Hospital (2020-YS-089) and registered in the Chinese Clinical Trial Registry with the identifier ChiCTR2000032430. All procedures with humans were performed in accordance with the ethical standards of the National Research Council. Following the Declaration of Helsinki, the researchers explained the significance of the study to all participants, who gave written informed consent. All medical records were anonymized, and no participant information was extracted for any reason except for the purposes of the study.

### Animals and Drugs

The *Tmem63a*^-/-^ mice and C57BL/6J mice were from Cyagen Biosciences. NPY-cre (Stock No. 027851) and Ai3 mice (Stock No. 007908) were from The Jackson Laboratory. Mice were group-housed and bred in Fudan University animal facilities with a 12 h /12 h light/dark cycle at 22 ± 1°C and free access to food and water. No statistical methods were used to predetermine sample size. No randomization was applied to the animal experiments. Sample sizes were chosen based on our previous studies on similar tests [45, 46]. All the animal procedures were approved by the Animal Care and Use Committee of the Institutes of Brain Science of Fudan University and were conducted in accordance with the National Institutes of Health Guide for the Care and Use of Laboratory Animals.

CFA, PTX, and formalin were from Sigma. The control and clodronate liposome were from Yeasen Biotechnology (Shanghai) Co., Ltd. IL-1β neutralizing antibody and control antibody were from BioXcell.

### Behavioral Tests

All mice for behavioral tests were habituated in a testing box (8 × 8 × 20 cm^3^) which was placed on the metal meshwork 2 days before testing.

#### Randall-Selitto Test

The Randall-Selitto test was conducted with Panlab apparatus by applying uniform increasing pressure on middle part of the tail to assess the threshold response to pain stimuli, and the pressure when the mouse flicked its tail to avoid it was recorded. The intensity of pressure causing an escape reaction was defined as the withdrawal threshold.

#### Tail Flick Test

Each mouse in the tail-flick test was fixed in a holder, then the tail was straightened and one third of the tail was immersed in a water bath at a set temperature. The time from immersion to a tail-flick was recorded as the latency. Three temperatures were tested (48°C, 50°C, and 52°C) and the cut-off times were set at 25 s, 15 s, and 10 s, respectively, to avoid tissue injury.

#### Hot-plate Test

The hot-plate test was conducted with a Bioseb cold-hot plate, and the time from placing the mouse on the plate to licking or flicking the hind paw was taken as the latency. The latencies at 50°C, 53°C, and 56°C were tested, and the cut-off times were set at 40 s, 30 s, 15 s, respectively, to avoid tissue injury.

#### Hargreaves Test

The Hargreaves tests were conducted with Hargreaves Apparatus (Ugo Basile). Mice were habituated in a testing box (8 × 8 × 20 cm^3^) which was placed on the glass panel 2 days before testing. 30% irradiation intensity was applied to the left hind paw, and the threshold was taken as the time when the irradiated hind paw was lifted or licked in response to the stimulus.

#### Formalin Tests

In the formalin tests, 10 μL of 5% formalin was injected into the left hind paw of mice by acute intraplantar injection. Pain behavior was recorded for 45 min with a Sony FDR-AX45\/AX60 camera. Videos were played back and the pain behavior was assessed in a double-blind manner. Lifting, licking, and flicking the hind paw were all taken as spontaneous pain behaviors. 0–10 min was taken as Phase I and 10–45 min was taken as phase II.

#### Mechanical Pain/Phantom Pain Test

To test mechanical sensitivity or phantom pain, we confined mice in behavioral testing boxes that were placed on an elevated metal mesh floor and stimulated their hind paws with a series of von Frey hairs with logarithmically increasing stiffness (0.16–2.00 g; North Coast Medical), perpendicular to the central plantar surface. We determined the 50% paw withdrawal threshold by Dixon’s up-down method [47].

#### Cold Pain Test

In the cold pain test, 30 μL acetone was gently applied to the bottom of a hind paw using a pipette through the mesh floor. Responses to acetone were graded on the following 4-point scale [46]: 0, no response; 1, quick withdrawal, flick, or stamp of the paw; 2, prolonged withdrawal or repeated flicking of the paw; 3, repeated flicking of the paw with licking directed at the ventral side of the paw.

#### Stump Pain Test

To test for stump pain, mice were confined in behavioral testing boxes which were placed on an elevated metal mesh floor. A trial targeting the connective tissue consisted of a train of 10 applications of a von Frey filament (0.16 g for 1–2 s) at 3–4 s intervals. A positive response was determined as a sharp withdrawal, shaking, or licking of the limb.

#### Adhesive Test

In the adhesive test, mice were placed on a transparent floor to habituate to the experimental environment for 15 min. On the experiment day, two paws of each mouse were stuck with an adhesive dot 6 mm in diameter. The response to the dots was recorded with a video camera for 15 min. Then the latency to removal of the sticky dots was recorded for each mouse.

### Paclitaxel (PTX) and Tibial Nerve Transfer (TNT) Mouse Models

To generate the PTX model, mice were anesthetized with isoflurane and then intraperitoneally injected with PTX in a single dose of 6 mg/kg, causing chemotherapy-related neuropathic pain. Mechanical allodynia and heat hyperalgesia appeared on day 3 after administration and lasted up to 28 days. The TNT model was generated following the procedure reported by Dorsi *et al.* [48]. Briefly, mice were anesthetized with isoflurane. After exposing the posterior tibial nerve from ~8 mm proximal to the calcaneal branch, we used 4–0 silk to tightly ligate the nerve and sharply transected it with scissors. Then, the silk pierced the skin at 3–5 mm superior to the lateral malleolus, where we tied two knots, and the stump was fixed underneath this location at the same time. The connective tissue was visible through the skin and served as the target for mechanical stimuli. Finally, the wound was carefully sutured. Three days later, we started to apply the behavioral tests.

### Macrophage Ablation

Wild-type mice were subjected to TNT surgery. 5 or 14 days later, these mice were grouped based on their pain threshold on day 5 and day 14. 10 μL of 5 mg/mL clodronate liposomes or an equal amount of control liposomes was administrated intrathecally. Pain behaviors were tested 24 h later, and then the DRGs of L4–L6 segments were dissected for subsequent tests.

### RT-PCR and qRT-PCR

Mouse tissues were rapidly isolated under RNase-free conditions. Total RNAs were extracted using TRIzol (Sigma, T9424). RNAs (0.5–1 μg) were reverse-transcribed using 5 × All-in-one RT MasterMix (abm). The sequences of the primers were 5’-GGACTCGCTGGTCAGGAAAG-3’ and 5’-CCCAGACACTAGGGGAAGGA-3’ for testing the mRNA levels of *TMEM63A* in human samples using qRT-PCR; and 5’-AGGCTGGGAGCATCTAGGAA-3’ and 5’-CCCATCAACCAGAAGTCCCC-3’ for testing the mRNA levels of *Tmem63a* in tissues from wild-type (WT) mice and *Tmem63a*^*-/-*^-knockout mice using RT-PCR. 5’-AGGCTGGGAGCATCTAGGAA-3’ and 5’-CCCATCAACCAGAAGTCCCC-3’, 5’-CCCCTCAGCAAACCACCAAG-3’ and 5’-CTTGGCAGATTGACCTCAGC-3’, 5’-GAAATGCCACCTTTTGACAGTG-3’ and 5’-TGGATGCTCTCATCAGGACAG-3’, 5’-AGGATACCACTCCCAACAGACCT-3’ and 5’-CAAGTGCATCATCGTTGTTCATAC-3’ for testing the expression levels of *Tmem63a*, *Tnf-α*, *Il-β*, and *Il-6*, respectively, in mouse tissue using qRT-PCR; 5’-GCGAGATCCCTCCAAAATCAA-3’ and 5’-GTTCACACCCATGACGAACAT-3’ for human GAPDH, 5’-AGGTCGGTGTGAACGGATTTG-3’ and 5’-GGGGTCGTTGATGGCAACA-3’ for mouse *Gapdh*.

### *In situ* Hybridization (ISH) and Immunohistochemistry (IHC)

Mice were deeply anesthetized with isoflurane and perfused through the ascending aorta with PBS, followed by 4% paraformaldehyde in 0.16 mol/L phosphate buffer. DRG sections were cut at 14 μm on a cryostat. ISH was applied with an RNAscope™ Fluorescent Multiplex Assay kit and probe targeting mouse *Tmem63a* from Advanced Cell Diagnostics strictly following the suggested procedure. After finishing ISH, the sections were incubated overnight at 4°C with the following primary antibodies: anti-SP (guinea pig, 1:1000; Neuromics), anti-CGRP (rabbit, 1:1000, Sigma), anti-TH (rabbit, 1:1000, Millipore), and anti-NF200 (mouse, 1:1000, Sigma), followed by Cy3- or FITC-conjugated secondary antibodies (1:200; Jackson Immuno Research Laboratories Inc.) or FITC-conjugated IB4 (10 μg/mL, Sigma-Aldrich). Sections for immunostaining were incubated with anti-ATF3 (rabbit, 1:1000, Abcam), anti-CD68 (rat, 1:200, Biolegend), and anti-β-tubulin III (mouse, 1:1000; Chemicon) followed by Cy3- or FITC-conjugated secondary antibodies (1:200; Jackson ImmunoResearch Laboratories Inc.), or DAPI (1:1000; Invitrogen), or Nissl (1:1000; Invitrogen). Sections were mounted and examined under a Nikon fluorescence microscope or Olympus FV1000 confocal laser scanning microscopy.

### Statistical Analysis

Data presented as bar graphs indicate the mean ± SEM (standard error of the mean). Dots in bar graphs and boxplots represent individual values per mouse or per image; a horizontal line indicates the average. Statistical analyses were performed in Prism 6.0 or Prism 8.0. Significance levels are indicated as follows: ns, *P* >0.05; ^*^P <0.05; ^**^P <0.01; ^***^P <0.001; ^*#*^*P* <0.05; ^*##*^*P* <0.01; ^*###*^*P* <0.001, unpaired Student’s *t*-test, one-way ANOVA, or two-way ANOVA unless otherwise noted.

## Results

### TMEM63A Expression is Increased in the Neuromas of Patients

**Fig. 1:**
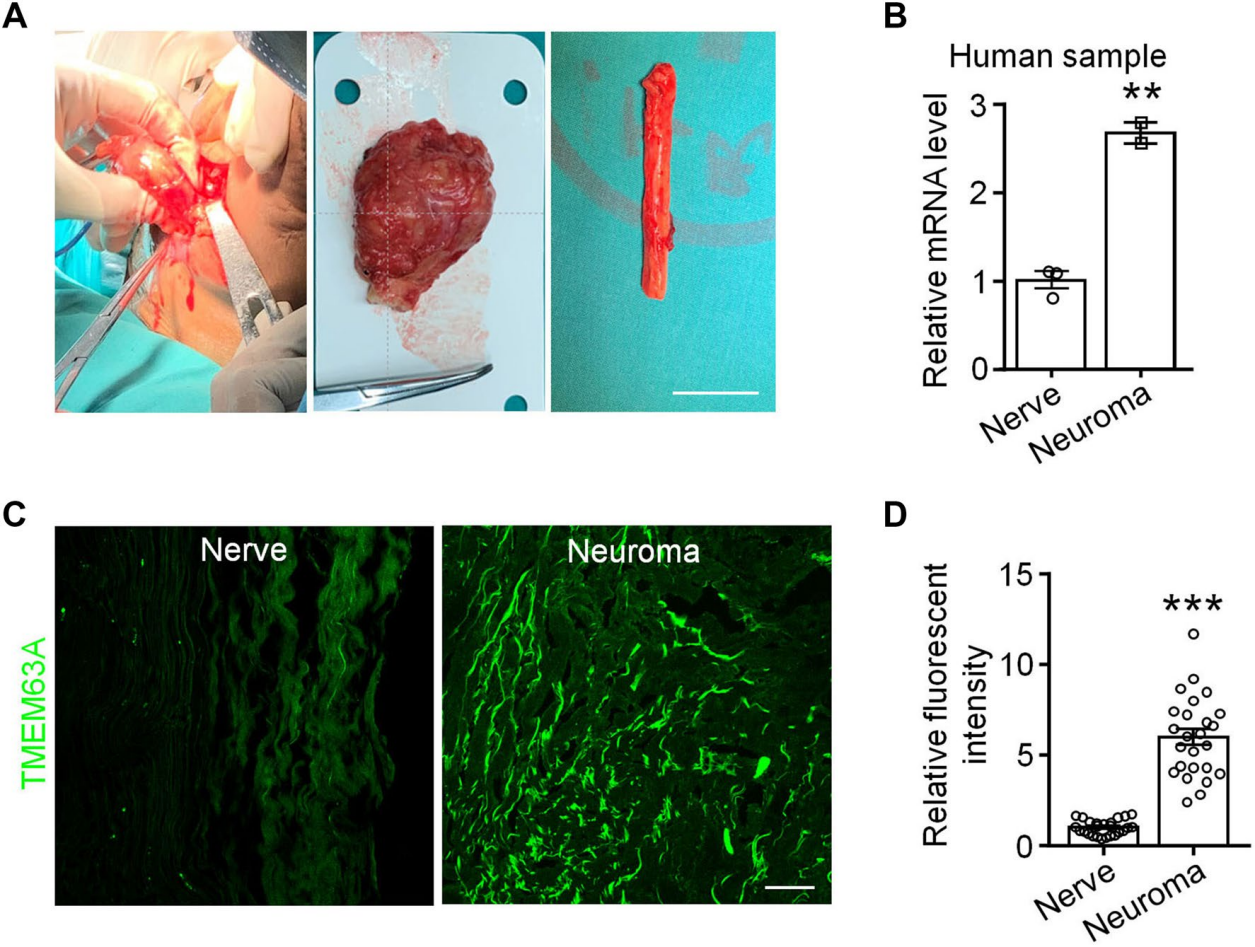
Expression of TMEM63A in human nerve and neuroma. **A** Representative images of a human nerve and neuroma. Left, image from the surgical procedure for dissecting the neuroma from an amputee; middle, the dissected neuroma; right, part of a human tibial nerve acutely isolated from a patient subjected to amputation due to a traffic accident (scale bar, 20 mm). **B** qRT-PCR results for *Tmem63a* expression in human tibial nerve and neuroma samples (*n* = 2–3, **P <0.01, unpaired Student’s *t*-test). **C** Immunostaining for TMEM63A with anti-TMEM63A primary antibody in the human nerve and neuroma tissue (scale bar, 200 μm). **D** Relative fluorescence intensity of TMEM63A in (**C**) (*n* = 24–25; ***P <0.001, unpaired Student’s *t*-test).

The three members of the TMEM63 family are TMEM63A, TMEM63B, and TMEM63C, all of which have been identified as mechanosensitive ion channels associated with osmotic pressure sensation in mammals [33]. We checked the expression of TMEM63 family members in the single-cell sequencing database [49, 50], and found that TMEM63A but not TMEM63B or TMEM63C is highly and specifically expressed in mechanical pain-producing non-peptidergic DRG neurons (http://linnarssonlab.org/drg/). Then we applied qRT-PCR to test the expression of *Tmem63a* in the neuroma tissue from patients suffering from chronic post-amputation pain and nerve tissue from amputees due to car accidents (Fig. 1A). This showed that the expression level of *Tmem63a* in neuromas was about double that in nerve tissues (Fig. 1B), suggesting a potential role of TMEM63A in the CPAPs. We also tested the expression of TMEM63A at the protein level by immunohistochemistry, and found that the fluorescent intensity of TMEM63A in neuromas was significantly higher than that in nerves dissected immediately from patients injured in car accidents (Fig. 1C, D), which was highly consistent with the qRT-PCR results (Fig. 1B). These results suggested that the expression of the mechanosensitive ion channel TMEM63A might be related to the formation of CPAP.

### *Tmem63a* is Specifically Expressed in Non-peptidergic Nociceptors

**Fig. 2:**
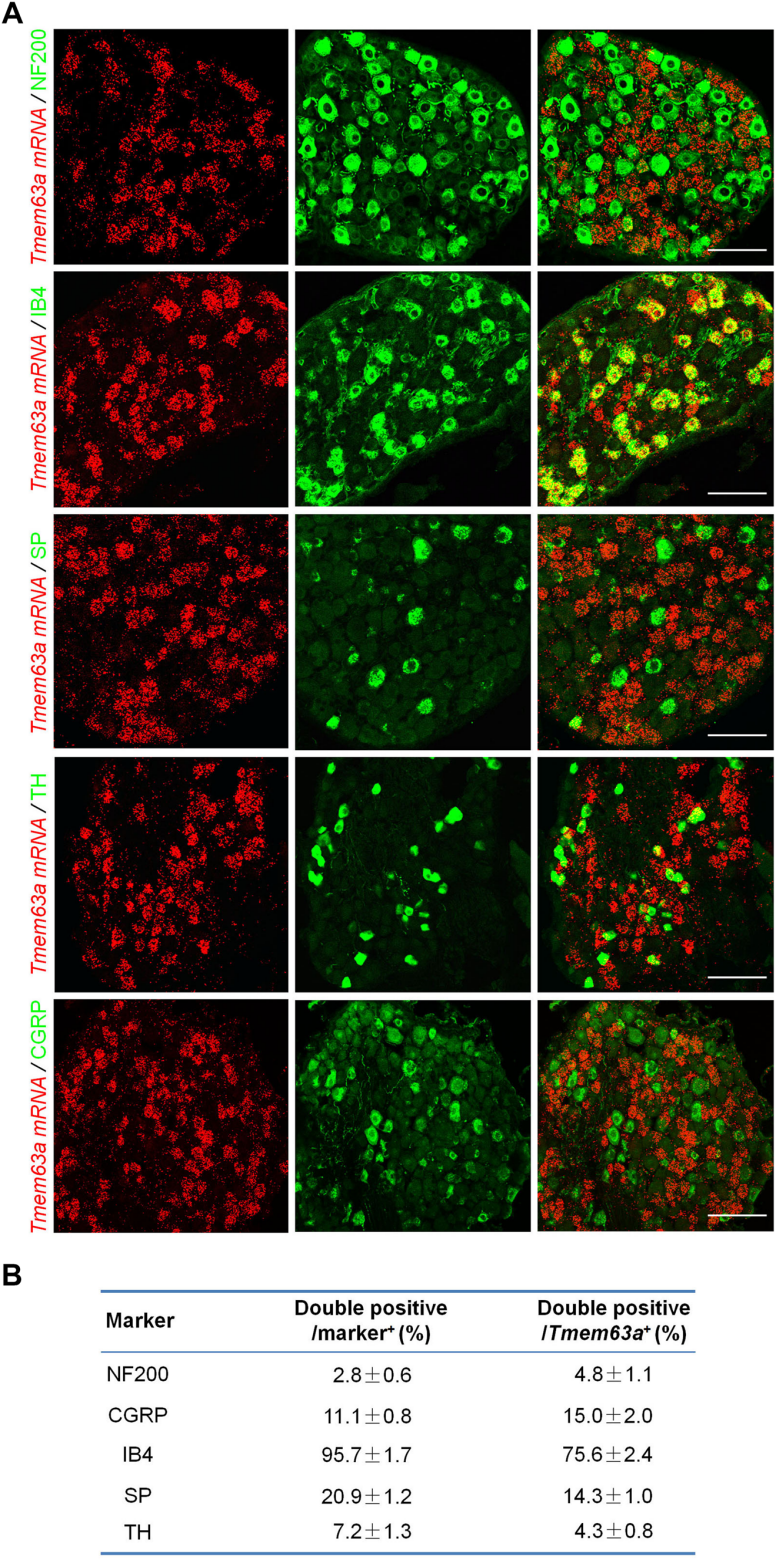
Cell-type profiling of *Tmem63a* expression in mouse DRGs. **A** ISH using a probe targeting mouse *Tmem63a* (red) combined with IHC to test the expression of *Tmem63a* in large DRG neurons (NF200), peptidergic DRG neurons (SP/CGRP), non-peptidergic DRG neurons (IB4), and C-fiber low-threshold mechanoreceptors (TH) (scale bars, 200 μm). **B** Cell-type profiling of *Tmem63a* in DRG neurons.

Then we characterized the cell-type profile of *Tmem63a* in the mouse DRG using ISH combined with IHC. The DRG is a heterogeneous structure consisting of a variety of cell types with versatile functions. Grossly, DRG neurons can be divided into NF200^+^ (neurofilament heavy chain-enriched) large-diameter low-threshold receptors, CGRP/SP (calcitonin gene-related peptide/substance P)-expressing peptidergic nociceptors, non-peptidergic nociceptors that label with isolectin B4, and TH^+^ (tyrosine hydroxylase) C-fiber low-threshold mechanoreceptors. The results showed a high level of *Tmem63a* expression in the DRG, which was mainly expressed in DRG neurons but not in satellite glial cells or macrophages (Figs 2A, S1). Specifically, 4.8% ± 1.1% of the neurons expressing *Tmem63a* were NF200^+^ large DRG neurons, 4.3% ± 0.8% expressed TH^+^ low-threshold mechanoreceptors, 14.3% ± 1.0% expressed the neuropeptide SP, 15.0% ± 2.0% expressed the neuropeptide CGRP, and 75.6% ± 2.4% were labeled with IB4, which indicated that *Tmem63a* is mainly expressed by non-peptidergic nociceptors (Fig. 2B). These findings strongly support the reliability of the single-cell RNAseq database released by the laboratories of Patric Enfors and Xu Zhang [49, 50]. We also analyzed the specificity of different subtypes of DRG neurons expressing *Tmem63a* and the results showed that it was expressed by 95.7% ± 1.7% of non-peptidergic nociceptors, 20.9% ± 1.2% of SP^+^ nociceptors, 11.1% ± 0.8% of CGRP^+^ DRG neurons, 7.2% ± 1.3% of TH^+^ DRG neurons, and 2.8% ± 0.6% of NF200^+^ DRG neurons (Fig. 2B). In addition, we applied ISH with a probe specifically targeting mouse *Tmem63a*, and combined it with IHC for the satellite glial cell marker glutamine synthetase. The results also showed that *Tmem63a* was specifically expressed in DRG neurons but not in satellite glial cells (Fig. S1A). We also purified peritoneal macrophages using fluorescence-activated cell sorting and used RT-PCR to test whether macrophages express *Tmem63a* in; the results showed that no *Tmem63a* expression was detectable in macrophages (Fig. S1B). Collectively, we conclude that *Tmem63a* is specifically expressed in DRG neurons.

These results showed that *Tmem63a* was specifically expressed in DRG neurons, mainly in mechanical pain-producing non-peptidergic neurons, and almost all of this subtype of nociceptors expressed the mechanosensitive ion channel TMEM63A.

### The Expression of TMEM63A is Increased in the Tibial Transposition Mouse Model of Neuroma

**Fig. 3:**
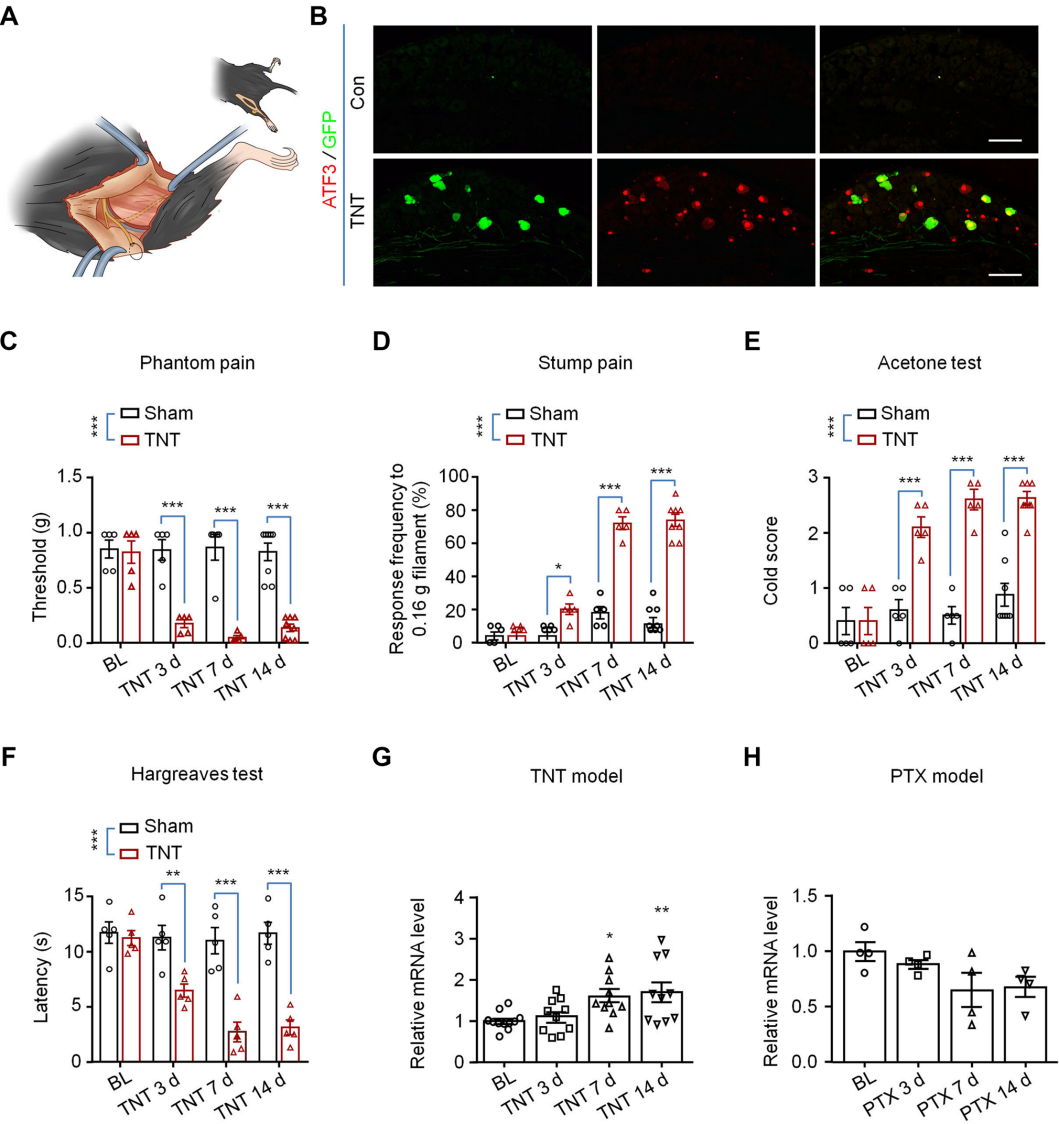
Generation and characterization of the TNT mouse model. **A** Schematic of the generation of the TNT mouse model. **B** IHC showing the expression of the neuronal injury marker ATF3 and NPY in NPY-Cre; Ai3 mice after TNT surgery (scale bars, 200 μm). **C–F** Behavioral tests of the TNT mouse model including phantom pain (**C**), *n* = 5–8, stump pain (**D**), *n* = 5–8, cold allodynia (**E**), *n* = 5–8, and heat hyperalgesia (**F**), *n* = 5. (*P <0.05, **P <0.01, ***P <0.001, two-way ANOVA). **G** qRT-PCR results for the expression of *Tmem63a* in the DRGs from TNT mouse models (*n* = 10–11; *P <0.05, **P <0.01, two-way ANOVA). **H** qRT-PCR results for the expression of *Tmem63a* in DRGs from chemotherapy (PTX) mouse models (*n* = 4; *P <0.05, one-way ANOVA).

In 2008, Dorsi *et al.* reported the TNT model in rats. The posterior tibial nerve of the TNT rat model was ligated and transected in the foot just proximal to the plantar bifurcation, which could mimic an amputation clinically [48]. Following the same procedure, we generated a TNT mouse model (Fig. 3A). Activating transcription factor 3 (ATF3) is a marker of stress-induced nerve injury, and neuropeptide Y (NPY) is a 36-amino-acid neuropeptide that is significantly upregulated after nerve injury [51]. In the TNT mouse model, we found that the expression of both ATF3 and NPY increased significantly (Fig. 3B); it has a pattern similar to that of the spared nerve injury mouse model [51]. We also found some of the TNT model mice formed swollen neuroma-like structures at the stump of the tibial nerve (Fig. 6A) 14 days after TNT surgery. Then we further tested the pain behavior of the TNT mouse models by stimulating the lateral part of the operated hind paw and the nerve stump separately with von Frey filaments to mimic phantom pain and stump pain, respectively. The results showed that strong mechanical allodynia developed 3 days after surgery, and the severe pain continued for 14 days or even longer (Fig. 3C, D). The TNT mouse model also developed severe cold allodynia (Fig. 3E) and heat hyperalgesia in the operated hind paw 3 days after surgery (Fig. 3F); this is comparable with the TNT rat model. Then we assessed the expression of *Tmem63a* in the DRGs of the mouse TNT model, and found it was significantly elevated (Fig. 3G), but not in the PTX-induced chemotherapy model (Fig. 3H), which was consistent with the data from the human neuroma sample (Fig. 1C, D). Collectively, all these data suggested the successful establishment of the TNT mouse model, and that MSC TMEM63A may play an essential role in post-amputation pain.

### Generation of *Tmem63a* Global Knockout Mice

**Fig. 4:**
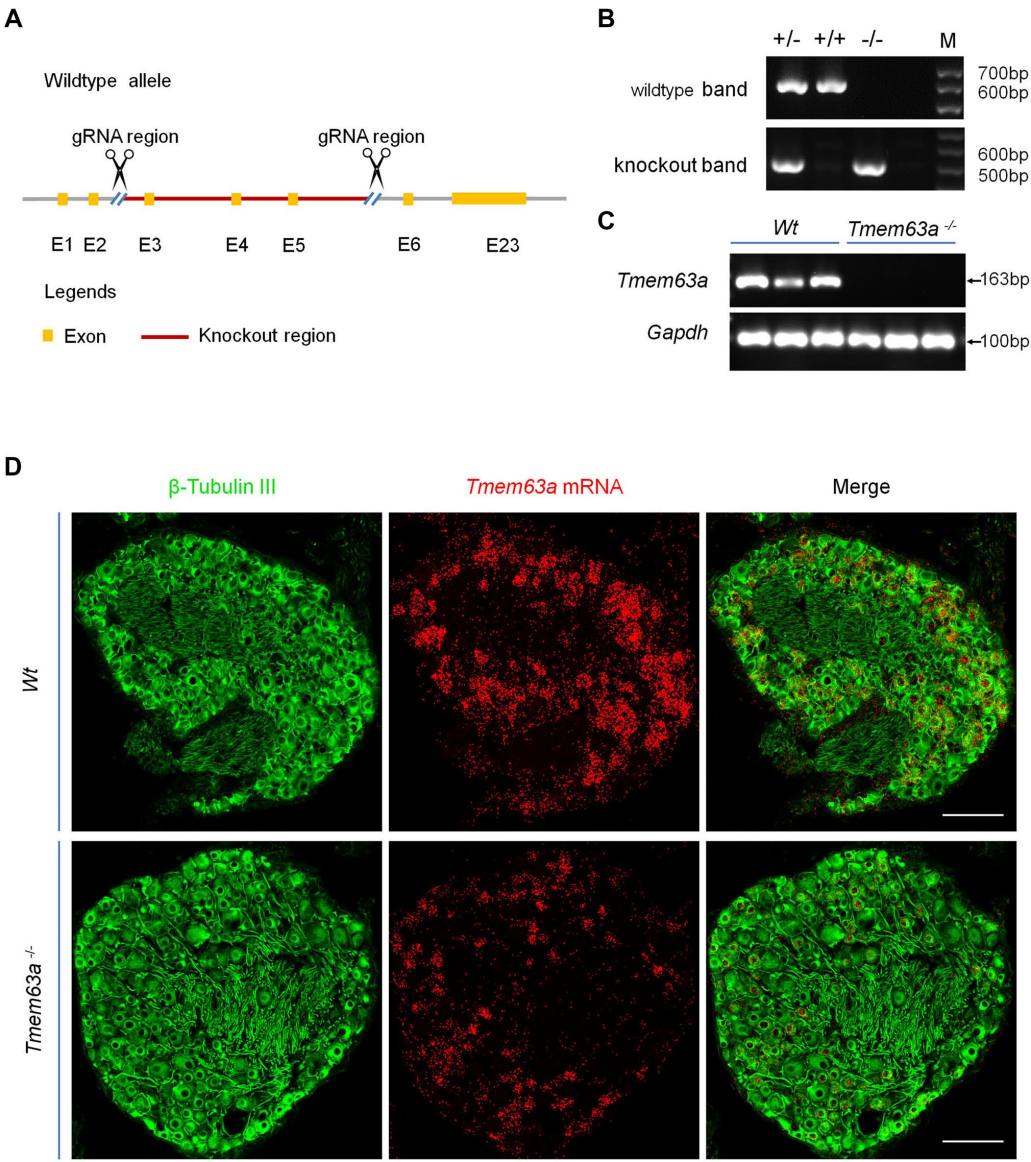
Generation of *Tmem63a* global knockout mice. **A** Schematic of the strategy for generating *Tmem63a* global knockout mice. **B** Genotyping of *Tmem63a*^–/–^ mice showing homozygous (*Tmem63a*^–/–^), heterozygous (*Tmem63a*^+/–^), and WT (*Tmem63a*^+/+^) mice with the wild-type band at 600 bp and the knockout band at 504 bp. **C** RT-PCR results for the knockout efficiency of *Tmem63a* in global knockout mice, three biological repeats per genotype. *Tmem63a* band at 163 bp and Gapdh band at 100 bp. **D** Representative images of ISH combined with IHC with pan neuronal marker β-tubulin III (green) to determine efficient knockout of *Tmem63a* (red) in DRGs (scale bars, 200 μm).

To test the role of TMEM63A in chronic pain, we generated *Tmem63a* global knockout mice with CRISPR-Cas9 technology, which deleted the coding region exons 3–5 of *Tmem63a* (Fig. 4A). We applied genotyping with primers annealing sites as indicated in Fig. 4A, and the results showed that both the WT band at 600 bp and the knockout band at 504 bp were present in the heterozygote, while only the WT band and the knockout band were present in the WT and homozygote separately (Fig. 4B). To further confirm the deletion efficiency in *Tmem63a*^-/-^ mice, we designed primers targeting the deleted region ranging from exon 3 to exon 5 and found that *Tmem63a* was completely abolished in the homozygote (Fig. 4C), confirming the successful generation of *Tmem63a*^-/-^ mice. Then we conducted ISH using RNAscope, and the result showed that the *Tmem63a* signal dramatically was decreased in the *Tmem63a*^-/-^ mice (Fig. 4D). However, since the ready-made probes of RNAscope contained multiple fragments targeting the entire mRNA from exon 1 to exon 23, exons other than exons 3–5 may have been transcribed and detected. Still, weak *Tmem63a* signals were still detectable by ISH. Collectively, these data indicated the successful generation of *Tmem63a* global knockout mice.

### TMEM63A is Essential for the Mechanical Pain Hypersensitivity in the Complete Freund’s Adjuvant (CFA) and TNT Models

**Fig. 5:**
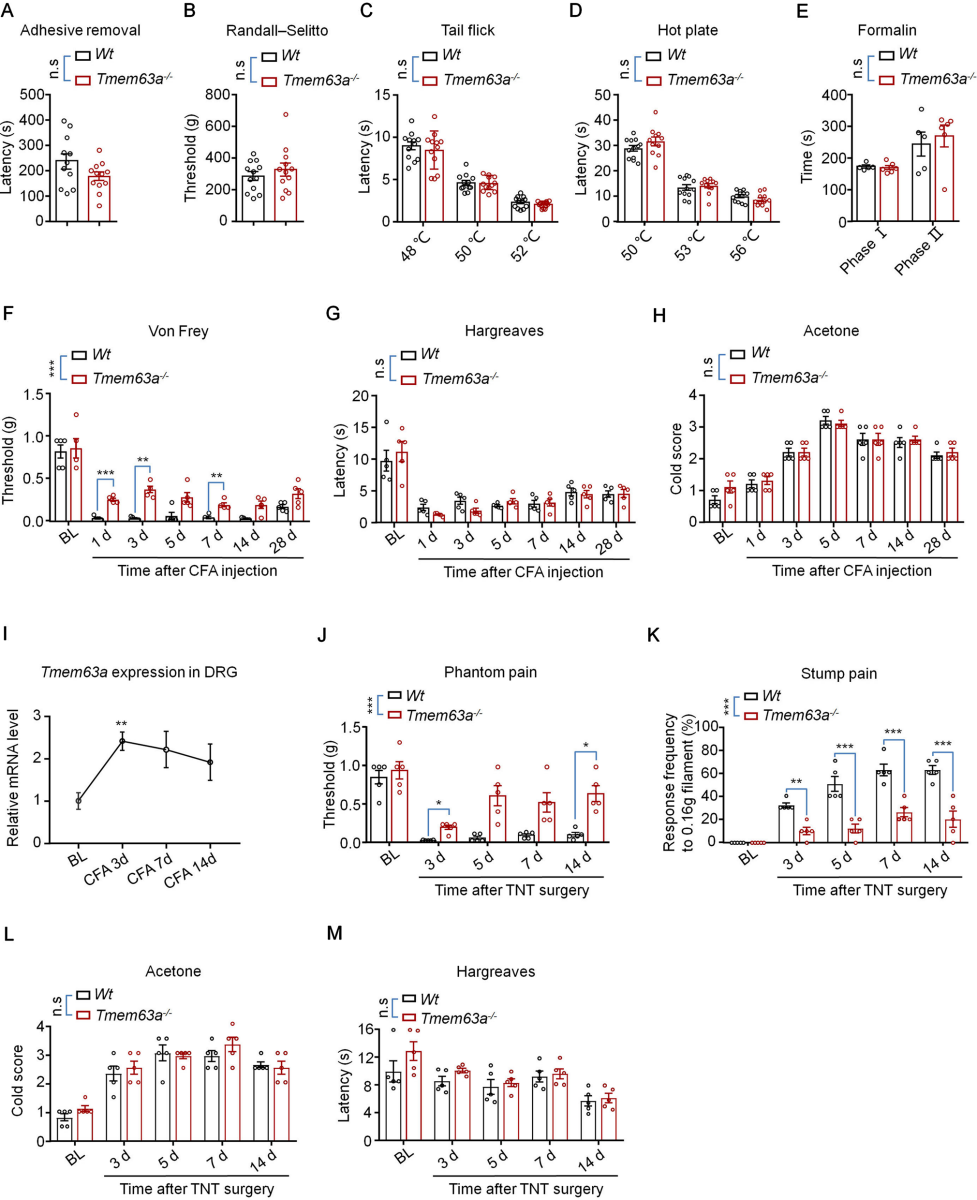
Effects of TMEM63A on touch sensation, basal pain, inflammatory pain, and CPAP. **A** Adhesive removal test checks the touch sensation of wild-type (WT) and *Tmem63a*^–/–^ mice (*n* = 11–12; n.s., *P* >0.05, unpaired Student’s *t*-test). **B** Randall-Selitto test evaluates nociceptive responses to deep mechanical stimuli of WT and *Tmem63a*^–/–^ mice (*n* = 12, n.s., *P* >0.05, unpaired Student’s *t*-test). **C** The tail flick test assays the reflex behavior of WT and *Tmem63a*^–/–^ mice to heat at 48°C, 50°C, and 52°C (*n* = 12; n.s., *P* >0.05, two-way ANOVA). **D** The hot plate test examines the response of WT and *Tmem63a*^–/–^ mice to heat at 50°C, 53°C, and 56°C (*n* = 11–12; n.s., *P* >0.05, two-way ANOVA). **E** Formalin test assesses the acute pain behavior of WT and *Tmem63a*^–/–^ mice after intraplantar injection of formalin (*n* = 5–6; n.s., *P* >0.05, two-way ANOVA). **F–H** The pain hypersensitivity of WT and *Tmem63a*^–/–^ mice in the CFA model: mechanical allodynia (**F**), heat hyperalgesia (**G**), and cold allodynia (**H**) (*n* = 5; n.s., *P* >0.05, ***P <0.001, two-way ANOVA). **I** qRT-PCR results for the expression of Tmem63a in the CFA mouse model (*n* = 3–4; **P <0.01, unpaired Student’s *t*-test). **J–M** Pain hypersensitivity of WT mice and *Tmem63a*^–/–^ mice in the TNT model: phantom pain (**J**), stump pain (**K**), cold allodynia (**L**), and heat hyperalgesia (**M**) (*n* = 5; *P <0.05, **P <0.01, ***P <0.001, two-way ANOVA).

Considering that TMEM63A is a mechanosensitive ion channel, we tested whether touch sensation is affected in the *Tmem63a*^-/-^ mice using the adhesive test, but no significant change was detected compared with WT mice (Fig. 5A). The Randall-Selitto test showed the deep pressure pain was not affected in the *Tmem63a*^-/-^ mice as well (Fig. 5B). The tail-flick and hot-plate tests showed that heat sensation was normal in *Tmem63a*^-/-^ mice (Fig. 5C, D). In addition, the spontaneous pain behavior induced by formalin was not affected in the *Tmem63a*^-/-^ mice (Fig. 5E). Together, these data suggested that basal pain sensitivity, i.e. normal pain perception and temperature sensation do not change in *Tmem63a*^-/-^ mice. Next, we investigated whether chronic pain was altered in *Tmem63a* mutant mice. Intraplantar injection of CFA induced persistent inflammatory pain for >2 weeks as characterized by mechanical allodynia, heat hyperalgesia, and cold allodynia (Fig. 5F–H). The mechanical allodynia but not the heat hyperalgesia and cold allodynia was significantly impaired in *Tmem63a*^-/-^ mice (Fig. 5F–H). Consistent with this, the expression level of *Tmem63a* also increased significantly on day 3 after CFA injection (Fig. 5I), indicating that the expression of *Tmem63a* in DRG neurons is associated with CFA-induced inflammatory pain. As expected, both phantom pain and stump pain improved after day 3 following TNT surgery in *Tmem63a*^-/-^ mice compared with WT mice (Fig. 5J, K). In sharp contrast, the heat hyperalgesia and cold allodynia were not affected in the *Tmem63a*-knockout mice (Fig. 5L, M). Overall, these data suggested that the mechanosensitive ion channel TMEM63A is specific and vital for the initiation and maintenance of mechanical pain hypersensitivity in both the CFA and the TNT mouse models.

### *Tmem63a* Ablation Reduces Macrophage Infiltration into the Residual Tibial Nerve and DRGs of the TNT Model

**Fig. 6:**
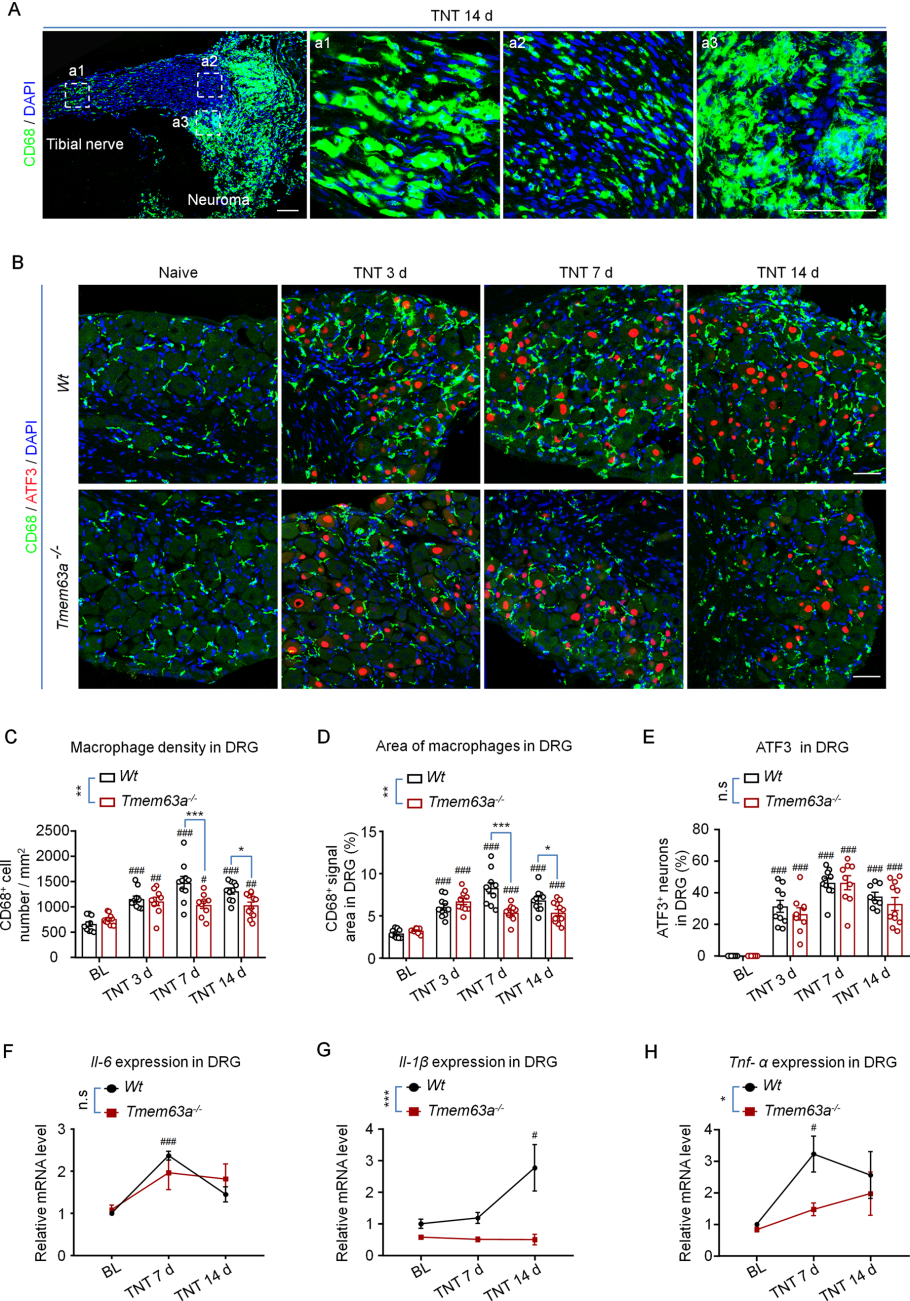
Macrophage infiltration and pro-inflammatory cytokine expression in the DRGs of *Tmem63a*^–/–^ mice. **A** IHC images of macrophage (green) infiltration in the tibial nerve and neuroma-like structure 14 days after TNT surgery; a1, a2, and a3 are enlargements of the boxed areas in the left (blue, nuclei stained with DAPI; scale bars, 50 μm). **B** Double immunostaining for the molecular markers for macrophages (CD68, green) and nerve injury (ATF3, red) in DRG sections from WT and *Tmem63a*^–/–^ mice after TNT surgery (blue, nuclei stained with DAPI; scale bars, 50 μm). **C** Quantification of macrophage density (cell number/mm^2^) in the DRGs from WT mice and *Tmem63a*^-/-^ mice after TNT surgery (*n* = 9–11; ^#^*P* <0.05, ^##^*P* <0.01, ^###^*P* <0.001, one-way ANOVA; *P <0.05, **P <0.01, ***P <0.001, two-way ANOVA). **D** Quantification of the average area of macrophages in DRG sections after TNT surgery (*n* = 9–12; ^###^*P* <0.01, one-way ANOVA; *P <0.05, **P <0.01, ***P < 0.001, two-way ANOVA). **E** Quantification of injured DRG neurons (*n* = 8**–**11; n.s, *P* >0.05, two-way ANOVA; ^###^*P* <0.001 one-way ANOVA). **F–H** qRT-PCR results for the expression of the pro-inflammatory factors *Il-6*, *Il-1β*, and *Tnf-α* in DRGs from WT mice and *Tmem63a*^-/-^ mice after TNT surgery (*n* = 3**–**4; n.s., *P* >0.05, **P <0.01, ***P <0.001, two-way ANOVA). In **C–H** ^#^*P* <0.05, ^##^*P* <0.01, ^###^*P* <0.001, baseline *versus* each time point, one-way ANOVA.

Innate immune cells, especially the macrophages infiltrating the DRG, play an essential role in both the initiation and maintenance of neuropathic pain [29]. To further investigate the mechanisms of how TMEM63A regulates CPAP, we checked whether the macrophage infiltration in the residual tibial nerve stump and DRGs of TNT model mice changed in the *Tmem63a*^-/-^ mice. The results showed that neuroma-like structures were formed at the residual site of the tibial nerve 14 days after TNT surgery (Fig. 6A). Simultaneously, there was strong macrophage infiltration in both the neuroma-like structures of axotomized tibial nerves (Fig. 6A) and DRGs (Figs 6B, S2) in the operated group. The density of macrophages in the DRG increased continuously from day 3 [(1150 ± 60)/mm^2^], peaked at day 7 [(1474 ± 122)/mm^2^], and was maintained until day 14 after the surgery [(1309 ±59)/mm^2^]; this was about twice that of naïve mice (Fig. 6C).

Meanwhile, the morphology of macrophages infiltrating the DRGs also changed, such as increased cell size and polarization (Fig. 6B) compared with naïve mice, indicating that macrophages are activated under TNT surgery conditions. Interestingly, the infiltration of macrophages decreased significantly in the DRGs of *Tmem63a*^-/-^ mice, while the expression of the nerve injury marker ATF3 did not change significantly (Fig. 6B–E), suggesting that the ablation of *Tmem63a* has an anti-inflammatory effect but not neuroprotective effect. We also tested the macrophage infiltration in the residual nerve stumps, and found no difference between the WT and *Tmem63*^*-/-*^ groups 7 days after TNT surgery (Fig. S3).

Then we checked the expression of the pro-inflammatory cytokines TNF-α, IL-1β, and IL-6 in the DRG of the TNT mouse model, and found that all the three increased markedly after TNT surgery in the DRGs of WT mice. The results showed that the expression of these cytokines were delicately orchestrated: *Il-6* and *Tnf-α* increased significantly at day 7 after surgery and decreased at day 14, while *Il-1β* increased dramatically only 14 days after surgery (Fig. 6F–H). We propose that *Il-6* and *Tnf-α* might be the principal cytokines responsible for the development of CPAP at the early stage, while the *Il-1β* are vital for the maintenance of CPAP. We also applied *Il-1β* neutralizing antibody by intrathecal injection into the TNT mouse model on day 14. The behavioral tests showed that both the phantom pain and stump pain were dramatically ameliorated after the administration of *Il-1β* neutralizing antibody (Fig. S5). All these data suggested that TMEM63A plays an essential role in regulating macrophage infiltration and the expression of pro-inflammatory cytokines in the DRG.

### Macrophage Infiltration Regulates the Expression of TMEM63A and CPAP

**Fig. 7:**
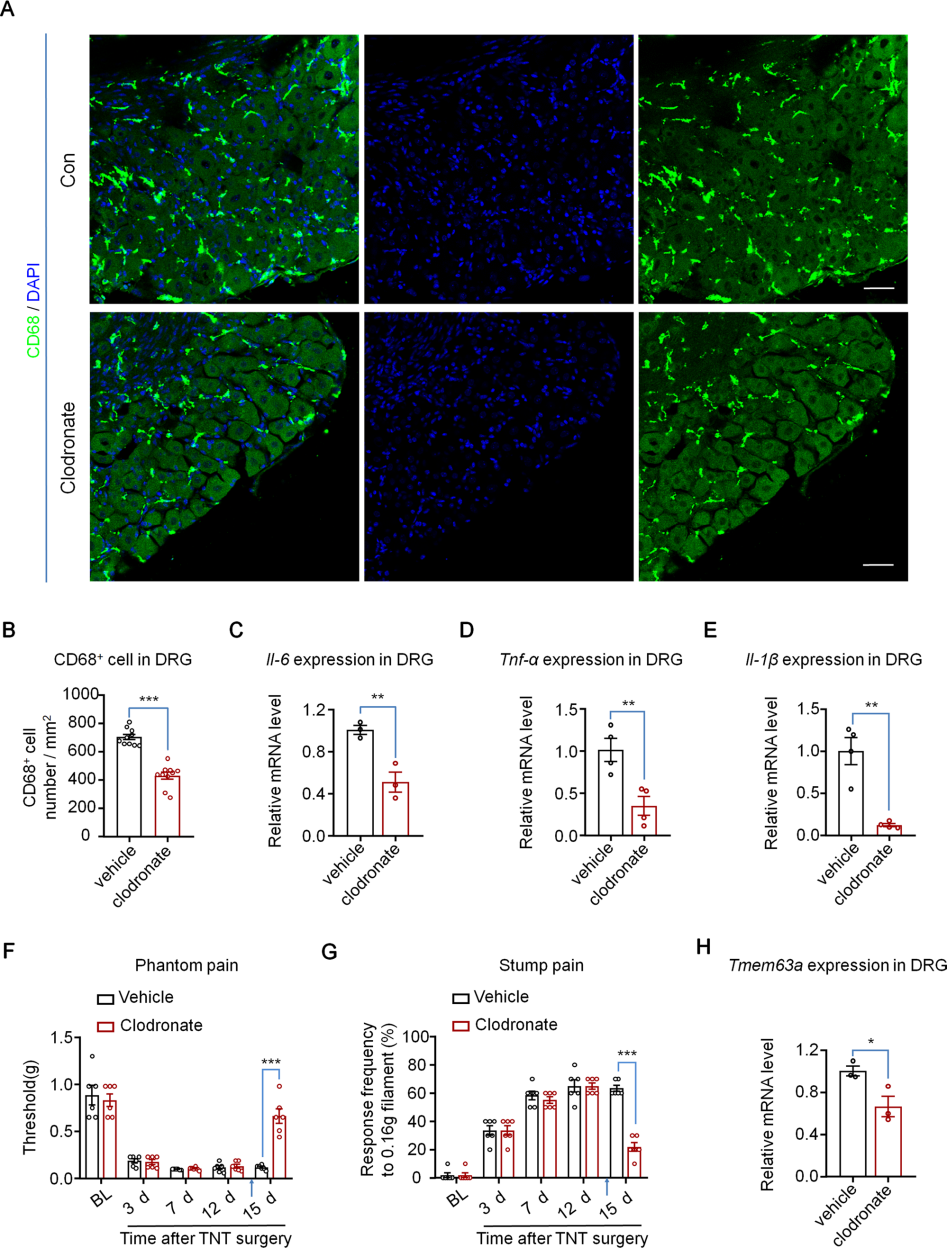
Pain behaviors, pro-inflammatory cytokines, and *Tmem63a* expression in DRGs after macrophage ablation. **A** Representative immunostaining of CD68 (green) in the vehicle and clodronate liposome groups (blue, DAPI stains nuclei; scale bars, 50 μm). **B** Quantification of macrophage ablation efficiency (*n* = 10; ***P <0.001, unpaired Student’s *t*-test). **C–E** qRT-PCR results for the expression of *Il-6* (**C**), *Tnf-α* (**D**), and *Il-1β* (**E**) (*n* = 3–4; *P <0.05, **P <0.01, unpaired Student’s *t*-test). **F–G** Pain hypersensitivity after macrophage ablation with clodronate liposomes. Both phantom pain (**F**) and stump pain (**G**) were tested 1 day after clodronate liposome administration (*n* = 6; ***P <0.001 unpaired Student’s *t*-test). **H** qRT-PCR results for the expression of *Tmem63a* (*n* = 3; *P <0.05, unpaired Student’s *t*-test).

Then we tested the effect of macrophage infiltration on CPAP and the expression of *Tmem63a*. Clodronate (dichloromethylene-bisphosphonate) is a hydrophilic molecule that can be encapsulated in liposomes. High cumulative concentrations of clodronate eliminate macrophages by initiating their programmed cell death [52]. We delivered clodronate liposome intrathecally into the mouse TNT model on day 5 or day 14, tested the pain behaviors 24 h later. We also checked the effects of ablation on macrophages and pro-inflammatory cytokine expression in the DRG obtained on naïve mice 24 h after clodronate injection. The results showed that the density of infiltrated macrophage in the DRG decreased significantly 24 h after clodronate administration (Fig. 7A, B). Consistent with this, the basal expression of IL-6, TNF-α, and IL-1β all decreased significantly in the DRG of naïve mice 24 h after clodronate administration (Fig. 7C–E). Subsequently, we examined the effect of clodronate treatment on pain behavior, and found that both the phantom pain and stump pain of day 5 and day 14 TNT mice were significantly reduced (Figs 7F, G, S4), which was also in line with a previous study [29]. Interestingly, the expression of *Tmem63a* also decreased significantly after eliminating the macrophages in DRGs (Fig. 7H). These data showed that macrophage infiltration is essential for the development of CPAP, and is also involved in the regulation of *Tmem63a* expression.

## Discussion

CPAP is still a vital clinical problem that tortures patients, but there are still no effective treatments are available due to the unknown mechanisms. To investigate the molecular mechanisms underlying CPAP, we generated a TNT mouse model that mimicked amputation clinically following the protocol reported by Dorsi on rats [48]. Using *Tmem63a*^-/-^ mice with exons 3–5 deleted and human neuroma samples, we made several interesting findings. First, *Tmem63a* was specifically expressed in DRG neurons, especially in the non-peptidergic nociceptors, and its expression was significantly increased in human neuroma and the DRGs of mice subjected to TNT surgery. Second, *Tmem63a*^-/-^ mice had marked deficits in inflammatory pain and neuropathic pain with predominant reductions in mechanical allodynia in the CFA model and TNT mouse model. Third, *Tmem63a* deficiency in DRG neurons impaired macrophage infiltration and pro-inflammatory cytokine expression in the DRGs from the TNT mouse model. Simultaneously, ablating macrophages decreased the expression of *Tmem63a*. Collectively, macrophage infiltration in the DRG is essential for the development of CPAP, and the deletion of *Tmem63a* significantly reduces macrophage infiltration in the DRG of the TNT mouse model and mechanical hyperalgesia in CPAP. This study discloses a novel mechanism for CPAP, which may provide a potential drug target for CPAP.

### Regulation of CPAP by the Mechanosensitive Ion Channel TMEM63A

CPAP is a long-lasting and severe pain after amputation surgery including both the stump pain arising from the residual part of the amputated limb [3] and the phantom pain arising from the denervated part of the limb [4, 5]. Undoubtedly, mechanical hyperalgesia is one of the most important components of CPAP both in the residual part of the amputated limb and the denervated part of the limb. Mechanosensitive ion channels are critical for the sensations of stretch, touch, vibration, proprioception, and pain [10]. Piezo2 is a typical mechanosensitive ion channel broadly expressed in DRG neurons; it is a stretch-gated ion channel that has been shown to be involved in mediating light touch, vibration detection, and proprioception. Loss-of-function mutations in Piezo2 completely fail to develop sensitization and painful reactions to touch after skin inflammation [19]. IB4-labeled non-peptidergic DRG neurons are critical for the generation of mechanical pain under physiological conditions, and TACAN expressed in this subset of DRG neuron is considered to be a potential mechanosensitive ion channel responsive to noxious mechanical force. Nociceptor-specific knockout of TACAN decreases the mechanosensitivity of nociceptors and reduces the behavioral responses to painful mechanical stimuli but not to thermal or touch stimuli [20]. TMEM63A is a newly-identified mechanosensitive ion channel. 75.6% ± 2.4% of *Tmem63a*^+^ DRG neurons were IB4^+^ DRG neurons and 95.7% ± 1.7% of IB4^+^ DRG neurons expressed *Tmem63a*, which marked the highly-specific expression of *Tmem63a* in non-peptidergic nociceptors (Fig. 2). Interestingly, the *Tmem63a*^-/-^ mice did not show any deficits in response to noxious mechanical stimuli under physiological condition (Fig. 5B, G), while the mechanical hypersensitivity but not heat hyperalgesia and cold allodynia were affected in the CFA mouse model and the TNT mouse model, suggesting quite different mechanisms for different pain modalities under physiological and pathological conditions. TMEM63A was particularly involved in mechanical pain hypersensitivity under pathological conditions such as inflammation and amputation.

The improved expression and elevated activity of mechanosensitive ion channels can lower the rheobase and threshold of mechanical nociceptors, which leads to the hypersensitivity of non-peptidergic nociceptors to mechanical stimuli and elicit pain [15, 19, 21]. Under CPAP conditions, the expression of MSC TMEM63A increased significantly in the neuroma and DRGs from TNT mouse models; the residual stump of the amputated tibial nerve was sensitized and more readily activated, which might lead to a reduced threshold of the mechanosensitive nociceptors and pain hypersensitivity.

The PTX model has been widely used to investigate the mechanisms of chemotherapy-induced neuropathic pain [53]. However, the expression of *Tmem63a* was not affected in this model (Fig. 3H), which is not consistent with that in the TNT and CFA models. This divergence might be due to pathogenic differences among the three models. It is still possible that TMEM63A also involved in regulating PTX-induced pain, because following the acute effect of PTX administration, it affects neuronal excitability, neurotransmitter release, and neuroimmune interactions [53], and all these activities are closely associated with the development of chronic pain.

### Regulation of CPAP by Macrophage Infiltration and Pro-inflammatory Cytokines

Macrophages are professional phagocytes responsible for the removal of dying or dead cells and cellular debris; they are mainly derived from circulating monocytes [54]. Increasing evidence has suggested that macrophages play an essential role in the initiation, maintenance, and resolution of chronic pain, depending on the location, stage, and phase of macrophages, respectively [25, 29, 30, 32, 55–57]. Furthermore, macrophages are also involved in the sex dimorphism of chronic pain through modulating the IL23/IL17A axis at the peripheral terminal ends of DRG neurons [57]. In this study, we found abundant macrophage infiltration into the DRGs of the TNT mouse model and the macrophages accumulated in the DRGs in a time-dependent manner (Figs 6B, C, S2). Meanwhile, the morphology of the macrophages that infiltrated the DRGs also changed, such as increased cell size and polarization (Fig. 6D), compared with that in naïve mice, indicating the activation of macrophages under TNT surgery conditions. At the same time, the expression levels of the pro-inflammatory cytokines IL-6,IL-1β, and TNF-α all increased (Fig. 6F–H). The constant and huge release of such cytokines might be critical for the formation of both stump pain and phantom pain. According to previous studies, macrophage infiltration into DRGs contributes greatly to both the initiation and maintenance of the mechanical hypersensitivity which characterizes the neuropathic pain phenotype [29]. The initiation and maintenance of phantom pain and stump pain were significantly reduced after ablation of macrophage at 5 and 14 days after TNT surgery (Figs 7F, G, S4). Furthermore, the expression of TNFα, IL-1β, and IL-6 increased with different time courses; TNFα and IL-6 increased 7 days after surgery and recovered soon, and IL-1β increased significantly 14 days after TNT surgery, indicating that the expression of these three pro-inflammatory cytokines were delicately orchestrated (Fig. 6F–H). It is quite possible that *Il-6* and *Tnf-α* might be vital cytokines for the development of CPAP at an early stage, while *Il-1β* is critical for its maintenance. The intrathecal injection of *Il-1β*-neutralizing antibody into TNT mouse models at day 14 strongly support this hypothesis. Therefore, the macrophage infiltration-induced pain in the TNT mouse model may be time-course-dependent due to the stage-specific expression of different cytokines (Fig. 6F–H). Quite different from blocking individual pro-inflammatory cytokines separately, ablating macrophage reduced the expression of all the three cytokines released by infiltrating macrophages. Finally, a vicious cycle was formed between nociceptors and DRG-resident macrophages under post-amputation or TNT surgery conditions, which was finely regulated by TMEM63A. TMEM63A potentiated the infiltration of macrophage and the expression of pain-related pro-inflammatory cytokines, which promoted the expression of *Tmem63a* and resulted in more macrophage infiltration and more severe CPAP.

Collectively, based on all the above data, we generated a working model of how the MSC TMEM63A modulates CPAP. After the amputation surgery, non-peptidergic nociceptors sense the injury and respond swiftly by elevating the expression of *Tmem63a*, which promotes the recruitment of macrophages into the injured tibial nerve. Higher pro-inflammatory cytokines released by infiltrated macrophages such as TNF-α, IL-1β, and IL-6 directly activate, sensitize nociceptors, and induce both acute pain and pain hypersensitivity [58], which results in severe CPAP. Furthermore, the increased macrophages and pro-inflammatory cytokines further promote the expression of *Tmem63a*, then promoting more macrophage infiltration and more severe CPAP. Disruption of this mutual interaction between macrophages and non-peptidergic nociceptor-expressed *Tmem63a* could effectively relieve the mechanical hypersensitivity components of CPAP, which provides a strong potential target for painkiller development.

## Supplementary Information

Below is the link to the electronic supplementary material.

Supplementary file1 (PDF 667 kb)
