# Supplementary material for: Mechanosensitive Ion Channel TMEM63A Gangs Up with Local Macrophages to Modulate Chronic Post-amputation Pain
Source: Neurosci Bull. 2022 Jul 12;39(2):177–93. doi: 10.1007/s12264-022-00910-0 (PMC9905372; doi:10.1007/s12264-022-00910-0)
Supplement: Supplementary file 1 — Supplementary file1 (PDF 667 kb) [file 12264_2022_910_MOESM1_ESM.pdf]

Supplementary Materials

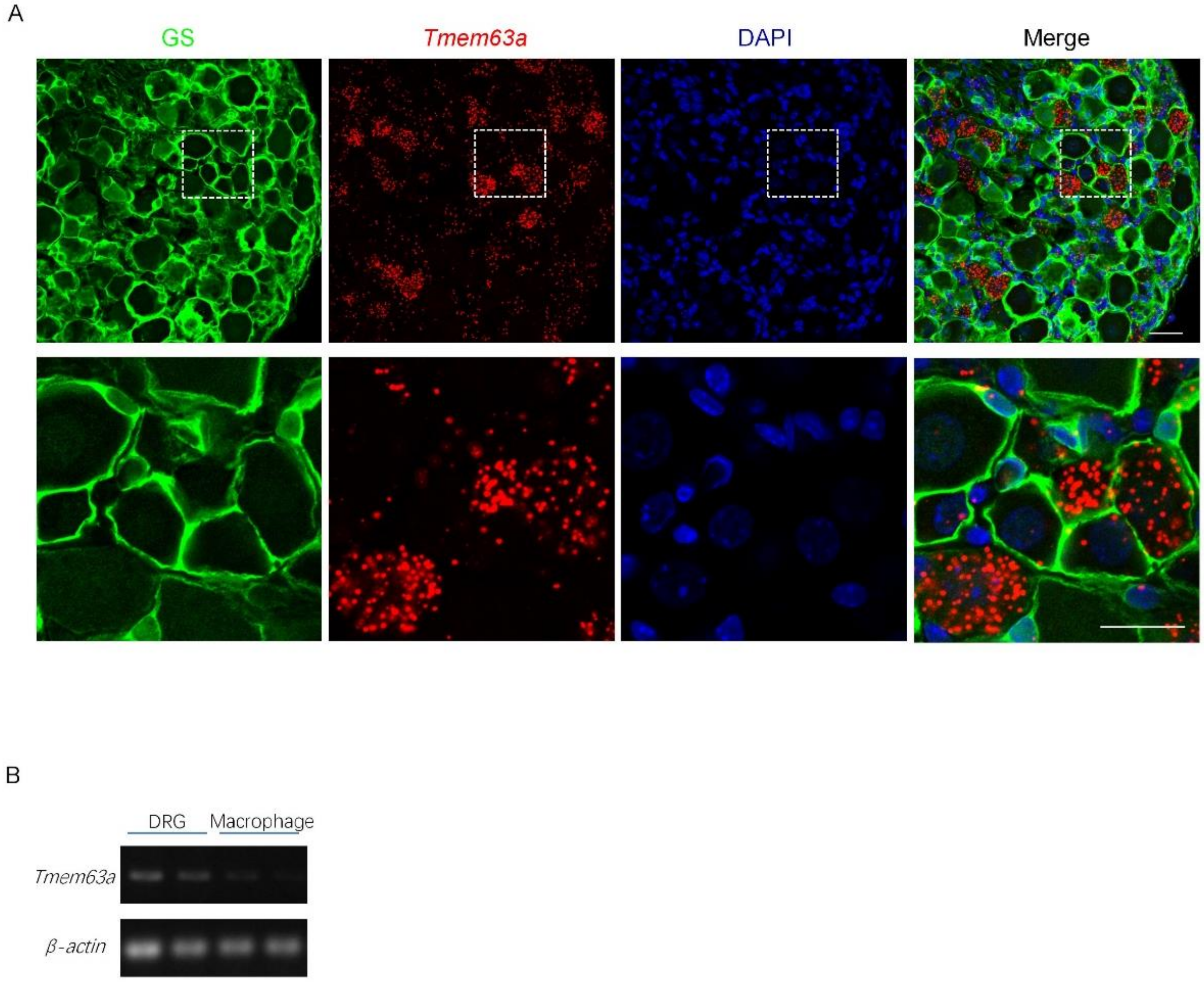

Fig. S1 The expression of TMEM63A in satellite glial cells and macrophages. **A** Representative images of ISH combined with IHC staining for the SGC marker GS (green) showing the expression of *Tmem63a* (red) in SGCs. The lower panels are partial enlargements of the boxed areas in the upper panels. Scale bars, 50 μm (upper) and 25 μm (lower). **B** RT-PCR images showing the expression of *Tmem63a* in FACS-purified peritoneal macrophages with CD68.

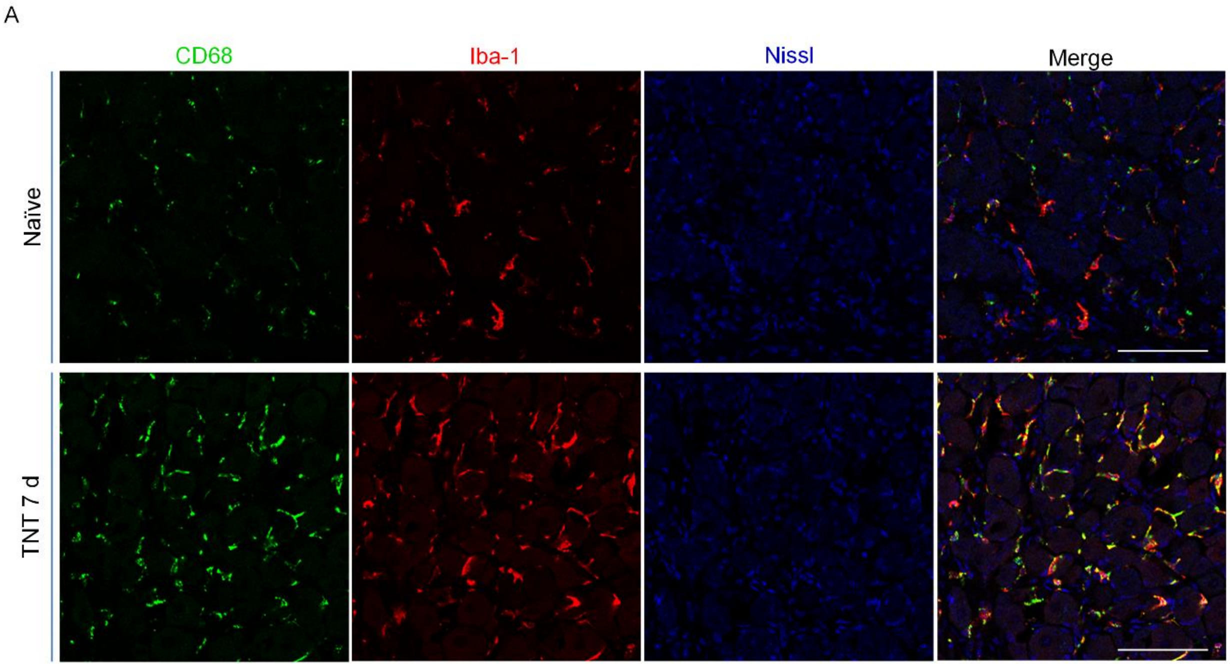

Fig. S2 Macrophage infiltration in the DRG of a naïve mouse and a TNT mouse. Double immunostaining with macrophage markers CD68 and Iba1 in the DRG from a naïve mouse and a day 7 TNT mouse (scale bars, 100 μm).

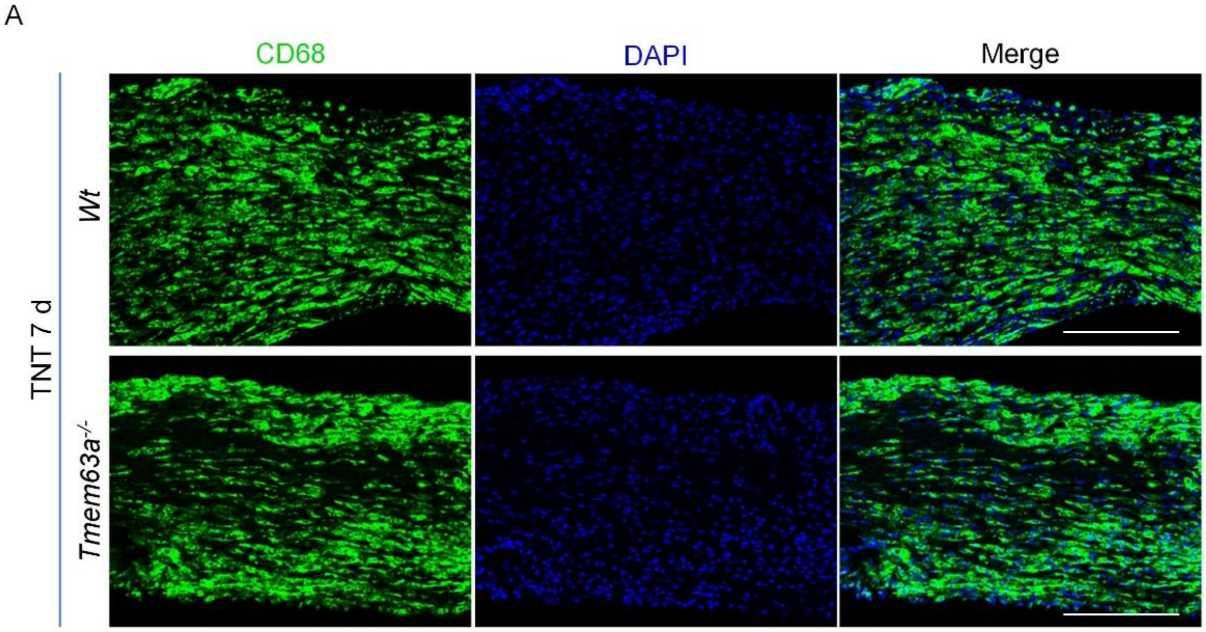

Fig. 3 Macrophage infiltration in the residual tibial stump of a WT and a *Tmem63a*$^{-/-}$ mouse. Immunostaining of CD68 in the residual nerve stump of WT and *Tmem63a*$^{-/-}$ mice on day 7 after TNT surgery (scale bars, 100 μm).

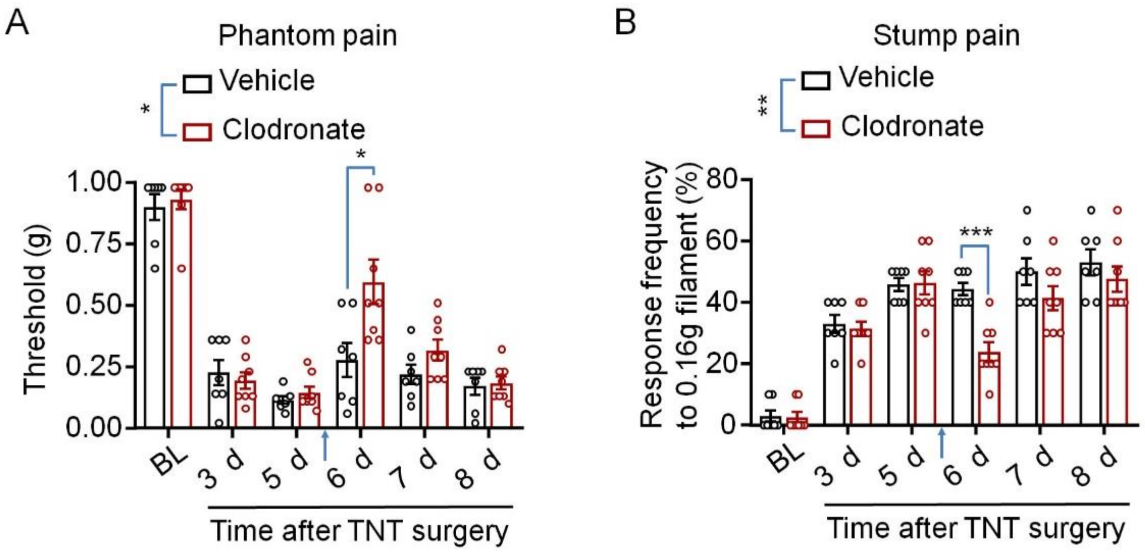

Fig. 4 The pain behaviors 24 h after macrophage ablation on day 5 after TNT. **A, B** Phantom pain (**A**) and stump pain (**B**) 24 h after intrathecal injection of clodronate liposomes on day 5 after TNT (blue arrows, time of intrathecal injection; n = 7–8; $^{*}P$ <0.05, $^{**}P$ <0.01, $^{***}P$ <0.001, two-way ANOVA).

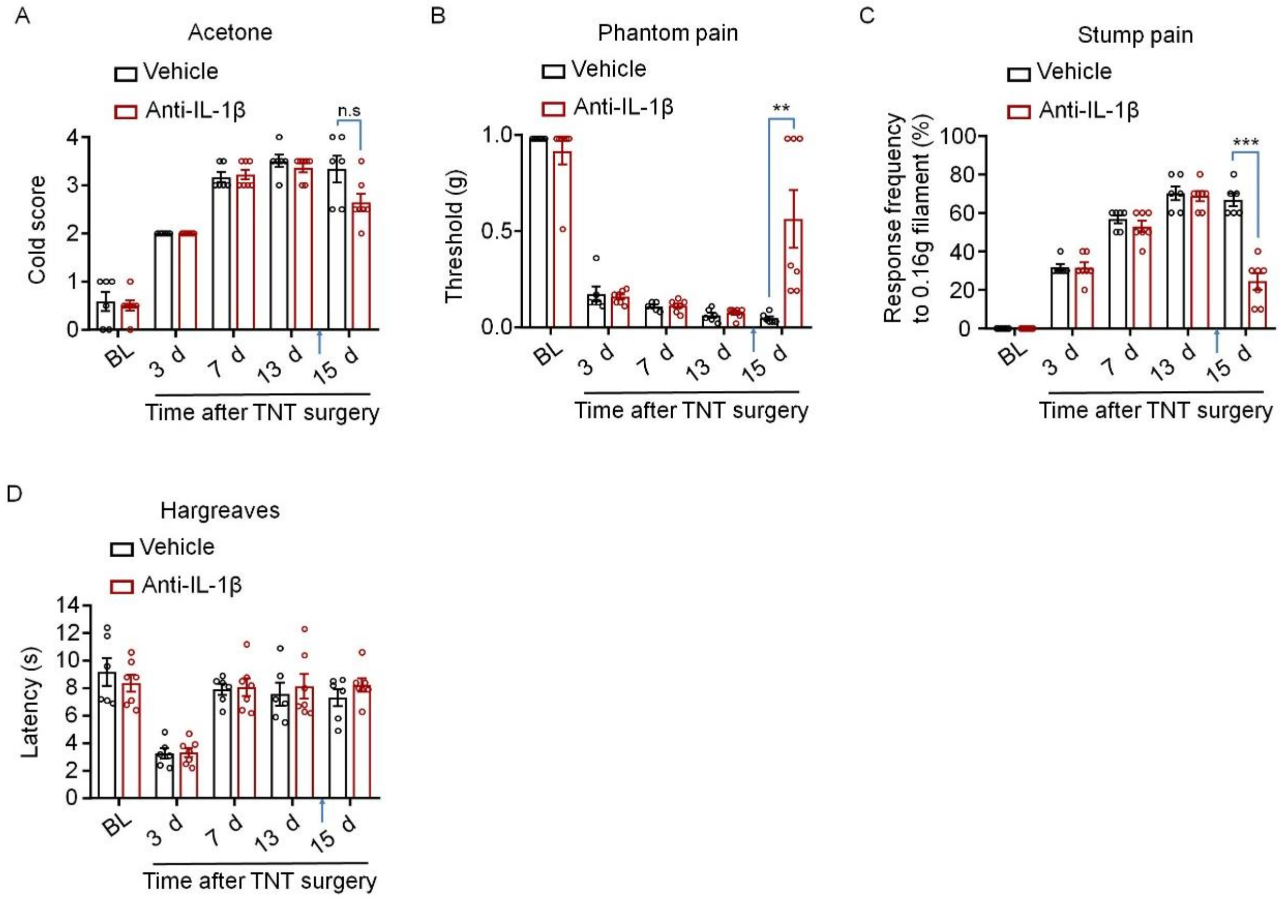

Fig. 5 The effect of IL-1β neutralizing antibody on cold allodynia (**A**), phantom pain (**B**), stump pain (**C**), and heat hyperalgesia (**D**) in the TNT mouse model. Mice were given control antibody or IL-1β neutralizing antibody (500 ng/μL, 10 μL in saline) 15 days after TNT surgery. Blue arrows, time of intrathecal injection; n = 6–7; $^{**}P$ <0.01, $^{***}P$ <0.001, two-way ANOVA.
